# In vitro estimation of superfluid critical extracts of some plants for their antimicrobial potential, phytochemistry, and GC–MS analyses

**DOI:** 10.1186/s12941-020-00371-1

**Published:** 2020-07-17

**Authors:** Waleed Bakry Suleiman

**Affiliations:** 1 Department of Botany and Microbiology, Faculty of Science, Al-Azhar University, The Permanent Camp St., 6th Ward, P.B. 13759, Nasr City, Cairo Egypt; 2 Faculty of Advanced Science and Technology, Kumamoto University, Kurokami 2-3-91, Kumamoto, 860-8555 Japan

**Keywords:** Phytochemistry, GC–MS, SFE, *Cichorium*, *Cinnamomum*, *Commiphora*, *Foeniculum*, *Nerium*, *Spartium*, Antimicrobial

## Abstract

**Background:**

Along with swift economic evolution and continuous amelioration of lifestyle, people at present are paying more attention to health issues. Synthetic drugs will be compensated with other natural ones that belong to natural origin. Plants have always been considered as sources of several compounds that are used in many fields, especially human and animal health, starting from boosting immunity to the treatment of infectious diseases caused by some pathogenic microbes such as bacteria, fungi as well as viruses. This study aimed to incorporate some types of plants within the antimicrobial portfolio through the examination of different six plants which were *Cichorium intybus*, *Cinnamomum camphora*, *Commiphora myrrha*, *Foeniculum vulgare*, *Nerium oleander*, and *Spartium junceum*. As well, attempting to identify the active constituents of their extracts using GC–MS.

**Materials and methods:**

All selected plants were analyzed to determine their phytochemical composition such as phenolics, alkaloids, flavonoids, terpenoids, and so on. The extraction step was done by sophisticated equipment called supercritical fluid extractor SFE through adjustment of specific conditions include temperature, time, flow rate and pressure to change the behavior of CO_2_. Testing the antimicrobial activity of each plant extract via agar well diffusion method through the formation of clear zones against a wide range of test microorganisms including both Gram-positive and Gram-negative bacteria as well as yeasts. Finally, attempting to primarily identify the constituents of each plant extract using GC–MS.

**Results and discussion:**

The crude extract of *F. vulgare* showed the highest potency against *C. albicans*, *E. faecalis* and *S. typhimurium*, it contains some unique compounds such as squalene, eugenol and isoeugenol while, Extract of *C. intybus* showed a moderate activity especially against *C. lipolytica* and MRSA and it includes Vitamin A like compound which indicates antioxidant property.

**Conclusion:**

Conclusively, fennel gave a promising result as a good wide spectrum antimicrobial agent because it contains some compounds act as antimicrobial agents such as eugenol which was used as food preservatives in addition to squalene which acts as an antioxidant and antimycotic agent so, it will be useful especially while it was used in highly purified form excluding all undesirable subcomponents.

## Background

The majority of microorganisms are free-living and perform useful activities however, some microorganisms caused diseases are called pathogens. They include bacteria, viruses, fungi, and protozoa. Infection occurs when a pathogen invades a body that resulted in clinical infection, such an infection is referred to as subclinical asymptomatic disease [1]. Microbial infections are most frequently caused by the resident microflora of the host rather than by exogenous invaders. Generally, harmless microorganisms may become virulent due to changes in the host’s tolerance or an alteration in the host’s microbial flora because of antibiotic use [2]. Antimicrobials reduced the morbidity and ameliorate human health against microbial infections. The incidence of antimicrobial resistance is increasing among all types of patients. Not all infections require specific antimicrobial treatment and careful clinical judgment is essential to determine whether symptomatic treatment is enough. Microbiological investigations should always be carried out before treatment [3]. Excessive use of antibiotics without advice of physicians, non-adjusted course of the antibiotic or uncompleted dosage, unsuitable antibiotic administration; all of these are the reason for bacterial resistance. Physicians should request a report of bacterial culture sensitivity (leastwise, as a phenotypic method) in cases of bacterial infection to detect the suitable antibiotic. Most of the *Staphylococcus aureus* isolates became MRSA (methicillin-resistant *S. aureus*), some of them became VRSA (vancomycin-resistant *S. aureus*), that means the scientists should act more and more to incorporate either more generations of antibiotics or new categories of antibiotics especially those belong to natural origin like plants, mushroom, truffles [4]. The use of plants in the pharmacological treatment of disease began long ago [5]. Centuries ago, Chinese, Japanese, and Indian used herbs in disease treatment as traditional medicine [6, 7]. In Europe and North America; herbal products have been expanded for use under many terms such as ‘alternative’, ‘complementary’, ‘holistic’ or ‘integrative’ medical systems [8].

*Cichorium intybus* was widely distributed in Africa, Asia-temperate, Asia tropical, Europe, Australia, Northern America, and Southern America. The phytochemical screening of *C. intybus* showed the presence of tannins, saponins, flavonoids, terpenoids, cardiac glycosides, and anthocyanins overall plant parts; seeds, leaves, and stem [9]. The *C. intybus* seeds extract contained appreciable levels of phenolic and flavonoid contents [10]. The flowers of chicory contain saccharides, methoxycoumarin, cichorine, flavonoids, essential oils, and anthocyanins [11, 12]. Unfortunately, no antimicrobial activity was reported against a wide range of microorganisms by using the hexane extract while; ethyl acetate did [13]. The root and leaf extracts (methanol, distilled water, chloroform, petroleum ether, and acetone) of *C. intybus* were investigated for antibacterial activity against Gram-negative pathogenic bacteria, the extracts showed a wide spectrum of inhibition against the test pathogens. Methanolic extract of the root and leaf proved to have the strongest antibacterial activity [14].

*Cinnamomum* spp. belong to Lauraceae and had been examined extensively for their essential oil constituents. This genus includes about 250 species in the tropical and subtropical districts, mostly in Asia and some in South and Central America, and Australia [15]. *Cinnamomum camphora* was known for its medicinal traits in folk medicine. Phytochemical screening showed a presence of alkaloids, Tannins, and carbohydrates, as well as its methanolic extract, presented the maximum antimicrobial activity when compared with the other extracts; chloroform and petroleum ether [16].

*Commiphora myrrha* belongs to Bruseraceae and it was commonly known as “Myrrh” which is one of the most important medicinal plants. Traditionally, its resin was used in the tackling of wounds, gastrointestinal tract GIT disorders, diarrhea, coughing, thoracic pain [17], gingivitis [18] also, it is very effective in the treatment of urinary tract infection UTI [19].

*Foeniculum vulgare* Mill is called fennel and belongs to Apiaceae, it features with its aromatic fruits. It was commonly used for the treatment of different disorders as well as it acts as a digestive, carminative, and diuretic agent [20]. Preliminary phytochemical screening confirmed the occurrence of flavonoids, tannins, saponins, steroids, glycosides, terpenoids besides its antimicrobial activity due to its potential essential oil constituents [21] as well as several pharmacological advantages through its bioactive constituents that are very important for human health [22].

*Nerium oleander* Linn; belongs to Apocynaceae family. It is commonly known as Kaner, an evergreen flowering shrub, and extensively cultivated for its aroma. Its extracts have many pharmacological properties such as a diuretic, expectorant, and sternutatory agent. It is also a highly toxic plant due to the presence of a cardiac toxin hence, it was used topically [23]. Methanolic and water extract of different parts of the plant gave a reasonable activity against some pathogenic microorganisms [24].

*Spartium junceum* is one of the medicinal plants that belong to the Fabaceae family that was cultivated as ornamental plant [25], its flowers were used for the treatment of gastric ulcers in Turkey [26] it has miscellaneous functions as antioxidant, antifertility, simulator for uterine and GI contraction thus causing vomiting [27–29].

The phenolic compounds could be separated by supercritical fluid extraction apparatus and this technique has been studied extensively 20 years ago [30]. Supercritical fluid extraction had been considered to attain the essential oil from *Psidium guajava* L. leaves, additionally, the non-volatile compounds were attained by CO_2_ supercritical extraction [31].

The current study aimed to screen the phytochemical composition of all mentioned plants in the review and extraction by a more sophisticated technique known as supercritical fluid extracting equipment SFE under certain conditions and subsequent investigation of the ability of these SFE extracts to inhibit the growth of some pathogenic microorganisms including Gram-positive and Gram-negative bacteria in addition to some pathogenic yeasts and finally, these SFE extracts would be analyzed by gas chromatography to predict their ingredients as an attempt to identify the bioactive components.

## Materials and methods

### Plants collected

Six different plants were collected from the local market and had been finely ground by aggressive blender until turning to a fine powder for easy extraction and further investigations, the following Table 1 includes their scientific, common and Arabic name as well as the parts used for investigations and analyses.

**Table 1:** Data of the plant samples that collected to be analyzed including the scientific, common, Arabic names in addition to the part of interest

Scientific name	Common name	Arabic name	Part of interest
Cichorium intybus	Chicory	Shikoryah-Hindbah	Seeds
Cinnamomum camphora	Camphor	Kafour	Leaves
Commiphora myrrha	Myrrh	Morr	Resin
Foeniculum vulgare	Fennel	Shamar	Seeds
Nerium oleander	Oleander-Kaner	Daflah	Flowers
Spartium junceum	Spanish broom	Wezal	Leaves

### Quantitative assessment for phytochemical constituents

Ten grams of the air-dried plant powder were extracted independently with 80% methanol three successive times. The extracts were concentrated, and the dried matter was then dissolved in 50 ml methanol. The alcoholic extracts were then completed to the volume of 100 ml by adding distilled water and used for the following determinations.

#### Total phenolics

One ml of the prepared extract of each specimen was completed to the volume of 10 ml by adding distilled water, then 1 ml of Folin–Ciocalteu reagent was added. The mixture was then shaken vigorously for 5 min, then 10 ml of 70% Na_2_CO_3_ was added and diluted immediately up to 25 ml by adding distilled water. The latter mixture was incubated for 90 min at room temperature. The absorbance was measured at 750 nm against the reagent used as a blank. A standard calibration plot was generated at 750 nm using known concentrations of gallic acid. The concentrations of phenols in the tested samples were calculated from the calibration plot and expressed as mg gallic acid equivalent of phenol/g of sample [32].

#### Total flavonoids

One ml of the prepared extract of each specimen was completed to the volume of 5 ml by adding distilled water. Immediately 0.3 ml 5% NaNO_2_ was added and the mixture was then left for 5 min. Respectively 0.3 ml 10% AlCl_3_ and 2 ml 1 M NaOH were added. The mixture was then diluted up to a volume of 10 ml with distilled water and the formed pink color was measured at 550 nm against the reagent used as a blank. A standard calibration plot was generated at 550 nm using known concentrations of rutin. The concentrations of flavonoids in the tested samples were calculated from the calibration plot and expressed as mg quercetin equivalent of flavonoids/g of the sample [33].

#### Total tannins

The total tannin content in the plant extract was estimated according to Folin-Deins reagent method [34], the absorbance was measured at 755 nm.

#### Total saponins

One gram of each plant powder was dispersed in 10 ml of 20% ethanol. Heating of the over a hot water bath for 4 h with persistent tilting at about 55 °C. The mixture was filtered, and the residue re-extracted with another 10 ml of 20% ethanol. The combined extracts were reduced to 2 ml over a water bath at about 90 °C. The concentrate was transferred into a 250 ml-separator funnel, and 5 ml of diethyl ether was added and shaken vigorously. The aqueous layer was retrieved whilst the ether layer was thrown away. The purification was done once again then 15 ml of *n*-butanol was added. The merged *n*-butanol extracts were washed twice with 10 ml of 5% aqueous sodium chloride, then the mixture was heated in a water bath. After evaporation, the samples were dried in the oven at 65 °C to a constant weight. The total saponin content was expressed as a percentage [35].

#### Total alkaloids

Ten grams of each plant powder was extracted with 90% ethanol. Mayer’s reagent was used according to Woo and Püls [36].

#### Total soluble carbohydrates

##### Extraction

According to the described method by Chaplin and Kennedy [37], the plant powder samples were dried at 65 °C till a constant dry weight and grounded again to a very fine powder by a clean mortar. One gram of the powder was put in a 100 ml conical flask, to which 5 ml of 2% phenol/water and 10 ml of 30% trichloroacetic acid were added. The mixture was shaken and kept overnight before being filtered; the filtrate was made up to 50 ml.

##### Estimation

Contents of total soluble carbohydrates were determined using anthrone technique [38], the developed color was measured using an electric colorimeter at 620 nm. A blank mixture containing distilled water and reagent was used to set up the apparatus at zero optical densities.

#### Total water-soluble proteins

##### Extraction

In this regard, one gram of the oven-dried powder at 60 °C and then transferred to 250 ml conical flask, then 10 ml distilled water, and 5 ml of 2% phenol solution were added. The contents of the flasks were shaken well and kept overnight before being filtered, and then they were used for the estimation of soluble proteins.

##### Determination

The optical density of the resulted color was then read at the wavelength of 750 nm. The concentration of soluble protein present in the sample was then calculated making use of the constructed standard curve of proteins [39].

#### Total nitrogen

The total nitrogen content of each plant powder was determined according to Kjeldahl digestion [40].

#### Volatile oil

Fifty grams of each plant powder was exposed to steam distillation to extract volatile oils according to Balbaa’s method [41].

### Extraction by supercritical CO_2_ fluid extractor (SFE)

Ten grams of each plant powder were used to be extracted via SFE equipment at SFE lab, the regional center for mycology and biotechnology RCMB, Al-Azhar University. The (Teledyne ISCO SFX 200) includes carbon dioxide injection pumps, extractors, separators, compressors, carbon dioxide tanks, chillers.

The operation of extraction depends upon pushing the CO_2_ through pipelines to be mixed with the samples under programmed critical conditions which gives CO_2_ the solvent property, its ability as a tunable solvent differs with changing the critical conditions, these parameters include pressure, temperature, flow rate. In this experiment; the conditions applied were 300 bar, 55 °C and CO_2_ flow rate of 6 g/min for 50 min.

### In vitro screening of antimicrobial activity of the plant SFE extracts

Agar well diffusion method described by [42] was applied to determine the antimicrobial activity of six extracts against 10 pathogenic microorganisms; the first four belonged to Gram-positive bacteria, the next four belonged to Gram-negative bacteria and the last two belonged to yeasts, the inoculum of each test organism was prepared according to [43]. This assay was done using gentamycin as a positive control antibacterial and ketoconazole as a positive control antifungal while methanol was selected roughly as a negative control. The medium used for bacterial growth was Mueller–Hinton agar medium (Code; CM0337, Thermo Scientific, Oxoid Microbiology products) which composed of g/l; beef dehydrated infusion 300, casein hydrolysate 17.5, starch 1.5 and agar 17, this medium is ready to use which had been prepared by weighing 38 g and up to 1 l with distilled water. On the other aspect, yeast was cultivated onto malt extract agar MEA medium (code; CM0059, Thermo Scientific, Oxoid Microbiology products) which composed of (g l); malt extract 30, mycological peptone 5, agar 15, this medium is ready to use which had been prepared by weighing 50 g and up to 1 l with distilled water. The test microorganisms were provided by the lab of antimicrobial and sensitivity assays, the regional center for mycology and biotechnology, Al-Azhar University, Cairo-Egypt, in which this experiment had been done.

### Determination of MIC and MBC/MFC for the most active plant extracts

Depending upon the clear zones diameters resulted by antimicrobial assay, out of the six plant extracts, two only were further investigated to determine their MIC values by which the lowest concentration of plant extract could inhibit the growth on the agar plate, MIC assay was accomplished by diluting the original crude extract (stock) through making these preparations (1:1, 1:2, 1:3, 1:4, 1:5, 1:6, 1:7). By the same protocol of agar well diffusion assay which applied to survey the antimicrobial activities of the six plant extracts, MIC also was determined and recorded. Consequently, MBC by which the minimum bactericidal concentration and MFC by which the minimum fungicidal concentration was determined according to [44] by transferring 10 µl from the non-turbid wells to the appropriate broth medium permitting the growth after overnight incubation and finally the turbidity would be determined by a spectrophotometer (UV/visible spectrophotometer; Milton Roy Spectronic 1201) at 530 nm wavelength according to [45].

### GC–MS analysis for prediction of subcomponents

GC–MS was carried out on Direct Probe Controller Inlet part to Single Quadrupole mass analyzer (Thermo Scientific; GC–MS model ISQ LT) using Thermo X-Caliber software. The Mass spectroscopy system was used to predict the subcomponents of plant SFE extracts using RTX-2330 (fused silica) 30 m capillary column of 0.25 µm internal diameter and df (µm) 0.20 µm. The column was operated at an initial temperature of 160–250 °C at the rate of 5 °C/min and was held for 30 min. The injector and detector temperatures were 240 °C and 250 °C, respectively. The carrier gas (nitrogen) was supplied at a total flow rate of 50 ml/min with a split ratio of 20:0 and subcomponents were identified by comparison with a linked library.

## Results and discussion

### Quantitative phytochemical screening

The powders of examined plants were processed according to the specific protocols to assess the phytochemical composition belongs to each plant, all the data obtained were tabulated in Table 2 which revealed the concentration of each component.

**Table 2:** Phytochemical composition of the six examined plants

Item	Item concentration for each plant					
	*Cichorium intybus*	*Cinnamomum camphora*	*Commiphora myrrha*	*Foeniculum vulgare*	*Nerium oleander*	*Spartium junceum*
Phenolics (mg EGA)	237	223	247	*261*	213	215
Flavonoids (mg ER)	143	138	*159*	131	124	118
Conc. %						
Tannins	*4.31*	4.13	4.05	3.87	3.74	3.27
Saponins	2.47	2.09	2.11	*2.69*	2.55	2.28
Alkaloids	*3.92*	3.85	3.49	3.85	2.99	2.87
Carbohydrates	2.15	*3.48*	3.07	2.21	2.86	2.94
Proteins	*15.94*	11.39	12.54	13.94	10.29	11.05
Total nitrogen	*26.76*	21.53	21.96	23.41	21.47	20.98
Oils	0.62	0.58	0.47	*0.81*	0.69	0.41

Italic values indicate the highest measurement among all items of the investigated plants

Phenolics were estimated in mg eq/gallic acid, flavonoids were estimated in mg eq/rutin while the rest of other items were estimated and expressed as percentages

Regarding total phenolic compounds; *F. vulgare* has the highest value of 261 mg followed by *C. myrrha* 247 mg, while the lowest value 213 mg belonged to *N. oleander*. In the case of flavonoid concentration; *C. myrrha* contains the highest value of 159 mg followed by *C. intybus* 143 mg, while the lowest concentration belonged to *S. junceum.*

Regarding tannins; the uppermost percentage went to *C. intybus* (4.31%) followed by *C. camphora* (4.13%) while *S. junceum* contains the lowest concentration of tannins (2.87%). Referring total saponin values; *F. vulgare* contains the highest percentage (2.69%) followed by *N. oleander* (2.55%) while the lowest percentage (2.09%) belonged to *C. camphora*.

Concerning alkaloids concentrations, the highest value belonged to *C. intybus* (3.92%) followed by both *C. camphora* and *F. vulgare* (3.85%) while the lowest percentage belonged to *S. junceum* (2.87%).

In the case of total soluble carbohydrates; *C. camphora* possessed the highest value (3.48%) followed by *C. myrrha* (3.07%) while the lowest carbohydrates content belonged to *C. intybus* (2.11%). Regarding soluble proteins and total nitrogen content, the highest value belonged to *C. intybus* (15.94% and 26.76% respectively) and followed by *F. vulgare* (13.94% and 23.41% respectively) while, the lowest percentage of proteins belonged to *N. oleander* and the lowest nitrogen content belonged to *S. junceum*.

Finally, in the case of oil percentage; the highest concentration was detected in *F. vulgare* (0.81%) followed by *N. oleander* (0.69%) while, the minimum value was detected in *S. junceum* (0.41%).

Regarding phytochemical screening, all examined plant are rich in phenolics, flavonoids, alkaloids, saponins, tannins and this is the reason of their pharmacological properties as good medicinal plants especially, fennel, chicory, and myrrh, this result is in harmony with anthocyanin that had been from red chicory *C. intybus* in aqueous solution at pH 2.5 [46].

Also, the current results of myrrh are compatible with those who recorded the presence of terpenoids, steroids, tannins, volatile oils, and resins [47].

**Fig. 1:**
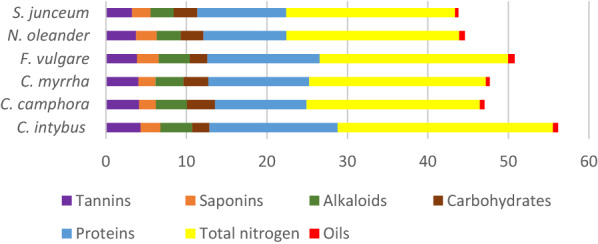
A stacked column chart expressing the values of phytochemical compounds among all examined plant samples measured in %

All data from Table 2 except total phenolics and total flavonoids (only phytochemical constituents that had been expressed in %) were configured in a stacked column chart (Fig. 1). By the first sight to Fig. 1, it could be noticed that *C. intybus* had the higher content followed by *F. vulgare*, *C. myrrha*, *C. camphora*, *N. oleander*, and the lowest plant content is *S. junceum*.

### Supercritical fluid extracting system

By applying selected parameters, the extraction capacity ranged from 0.4 to 0.6 ml which represents 4–6 wt% approximately (the original concentration of the stock crude extract). The advantage of using this technique for extraction is a high precision, time-saving, and no solvent traces. This result expressed success of SFE equipment in extraction process; this is friendly with the results reported that the use of SFE in last years had been approved to be alternative for extraction of natural compounds like triterpenes which extracted by both SFE in corresponding to traditional extraction method; Soxhlet and the result of SFE was more satisfying [48]. As well, SFE is a green extraction method providing a concentrated end product with no undesirable residues [49]. The effect of SFE parameters changing to obtain better extractability of phenolic compounds from lavender flowers, different ranges of SFE parameters were checked such as 200 bar, 40 °C and 15 min and the yield was 4.3 wt% while 250 bar, 60 °C for 45 min gave 9.2 wt% (more than double) [50].

### Antimicrobial activity determination

Antimicrobial activity of these six different plants was estimated, and the results had been expressed in mm as diameters of inhibition zones. All the data resulted were tabulated in Table 3 which revealed the following observations:

**Table 3:** Antimicrobial activity of SFE plant extract against some variable pathogenic microorganisms

Test organism	*Cichorium intybus*	*Cinnamomum camphora*	*Commiphora myrrha*	*Foeniculum vulgare*	*Nerium oleander*	*Spartium junceum*	Positive control
Pathogenic yeasts Ketoconazol 100 µg (positive control)							
*Candida albicans* RCMB 005003 (1) ATCC 10231	–	11	–	14	–	12	20
*Candida lipolytica* RCMB 005004	18	16	17	15	10	8	18
Gram-positive bacteria Gentamycin 4 µg (positive control)							
MRSA clinical isolate	11	–	7	8	–	–	30
*Enterococcus faecalis* ATCC 29212	18	12	13	22	10	13	26
*Streptococcus mutans* RCMB 017 (1) ATCC 25175	–	–	–	–	–	–	20
*Micrococcus* sp. RCMB 028 (1)	–	–	–	–	–	–	22
Gram-negative bacteria Gentamycin 4 µg (positive control)							
*Enterobacter cloaca* RCMB 001 (1) ATCC 23355	–	–	–	–	–	–	27
*Klebsiella pneumonia* RCMB 003 (1) ATCC 13883	–	–	–	–	–	–	–
*Proteus vulgaris* RCMB 004 (1) ATCC 13315	–	–	8	7	–	–	25
*Salmonella typhimurium* RCMB 006 (1) ATCC 14028	14	16	11	20	–	–	17

Data expressed as diameters of the inhibition zones in mm ranged from 7 to 22 mm for the plant extracts in corresponding to the inhibition zones resulted by positive control which ranged from 17 to 30 mm

All test organisms exhibited variation in their responses against the examined crude extracts, *Candida albicans* exhibited more resistance than *C. lipolytica* among the examined yeast strains; only three extracts had moderate effects against *C. albicans* while all six extracts affected *C. lipolytica.*

In the case of Gram-positive bacteria; the most resistant strain was *S. mutans* followed by *Micrococcus sp.* and MRSA had been affected only by three extracts while *E. faecalis* was the most susceptible one that had been inhibited by all six investigated extracts.

Referring to Gram-negative bacteria; the most resistant strain was *K. pneumonia* followed by *E. cloaca* and *P. vulgaris* (only two extracts weakly inhibit its growth) while *S. typhimurium* was the most susceptible one that had been inhibited by four extracts.

On the other side, the investigated crude extracts might be categorized as potent, moderate, and weak antimicrobial agents according to the applied concentrations, also, some of them could be classified as wide spectrum while the others are narrow spectrum. For example; crude extract of *F. vulgare* showed the highest potency against *C. albicans*, *E. faecalis,* and *S. typhimurium*, it also showed a wide spectrum against seven out of 10. This result is in line with those who had investigated phytochemistry, antimicrobial activity and GC–MS of Portuguese fennel fruits that gave very compatible results with the current study where the crude extract has antimicrobial activity slightly lower than those belonging to the positive standard used; tetracycline as antibacterial and nystatin as antifungal [51]. Also, our finding showed a moderate effect of fennel extract against methicillin-resistant *Staphylococcus aureus* (MRSA) clinical isolate and this result is relative to those findings which concluded that the combination between fennel essential oil and mupirocin has a significant eradicating effect against *S. aureus* and his finding will be useful antistaphylococcal agent [52].

Extract of *C. intybus* showed a moderate activity especially against *C. lipolytica* and MRSA with moderate range against four out of 10, while the extract of *C. myrrha* gave a moderate to weak activity with wide spectrum against six out of 10. This result in a harmony with hexane and ethyl acetate extracts of chicory roots that showed pronounced antibacterial activity against both Gram-positive and Gram-negative bacteria [53].

SFE extract of *C. myrrha* has a moderate antimicrobial activity according to the experimental circumstances, it is only active against five out of 10. This result slightly related to the conclusion related to myrrh extract which has a noticeable antibacterial activity against *Enterococcus faecalis* and *Fusobacterium nucleatum* involved in root canal infections especially when it combined with sodium chlorite [54].

Finally, *S. junceum* and *N. oleander* could be classified as weak and narrow-spectrum antimicrobial agents; they only active against three and two out of 10 respectively. This finding is consistent with the previously published data that are very limited about antimicrobial activity of Spanish broom. Oleander leaves have antimicrobial activity against *Bacillus pumilus*, *B*. *subtilis*, *S*. *aureus*, *E*. *coli*, and *Aspergillus niger* [55].

### Determination of MIC and MBC/MFC of the plant extracts

According to the results of antimicrobial activity assay previously, the crude extracts of both of *C. intybus* and *F. vulgare* were chosen for further investigation for their values of MIC, MBC/MFC. The original concentrations of *C. intybus* and *F. vulgare* were 5 and 4% respectively. Different dilutions were prepared and investigated against the indicator strains via agar well diffusion assay, the data resulted was represented in Table 4. The MIC values for *C. intybus* against *Candida lipolytica*, MRSA, *E. faecalis* and *Salmonella typhimurium* were 1.25, 2.5, 1.67 and 1.67% respectively, clarifying that the most susceptible test organism is *C. lipolytica*, and accordingly MBC/MFC was determined, MFC of SFE extract of was 5% which was the original concentration and it was considered as the minimum fungicidal concentration against *C. lipolytica*, as well the MBC was 5% as the minimum bactericidal concentration against *E. faecalis* only, nonetheless the other susceptible bacteria were not completely killed so, the examined concentrations giving the clear zones were considered as bacteriostatic and these bacteria need a higher concentration to be killed completely.

**Table 4:** MIC, MBC/MFC determination for both chicory and fennel extracts

Test organism	Chicory			Fennel		
	Stock (mm)	MIC %	MBC/MFC %	Stock (IZ)	MIC %	MBC/MFC %
*Candida albicans* RCMB 005003 (1) ATCC 10231	ND	–	–	14	1.33	ND
*Candida lipolytica* RCMB 005004	18	1.25	5	15	1.33	4
MRSA clinical isolate	11	2.5	ND	8	4	ND
*Enterococcus faecalis* ATCC 29212	18	1.67	5	22	0.8	2
*Proteus vulgaris* RCMB 004 (1) ATCC 13315	ND	–	–	7	4	ND
*Salmonella typhimurium* RCMB 006 (1) ATCC 14028	14	1.67	ND	20	0.8	2

Stock represents the original concentration in corresponding to the inhibition zones diameters expressed in mm, while MIC and MBC/MFC describe the responsible concentration in %

On the other hand, the MIC values for *F. vulgare* against *C. albicans*, *C. lipolytica*, MRSA, *E. faecalis*, *Proteus vulgaris* and *S. typhimurium* were 1.33, 1.33, 4, 0.8, 4 and 0.8% respectively, clarifying that the most susceptible test organisms are *E. faecalis* and *S. typhimurium.* Accordingly, MBC/MFC was determined, MFC of SFE extract of was 4% against *C. lipolytica*, likewise, the MBC was 2% *E. faecalis* and *S. typhimurium*, nevertheless the other susceptible test organisms were not completely killed so, the examined concentrations giving the clear zones were considered as bacteriostatic and these strains need a higher concentration to be killed completely.

The entire results of MIC were in a harmony with [56] who reported variability of MIC values of *C. intybus* extracted by three different solvents against a limited number of test organisms. No much more information about the mechanism of action of antimicrobial agent(s) from *C. intybus* whether bactericidal or bacteriostatic. Regarding *F. vulgare*, the most valuable antimicrobial activity belongs to its essential oil neither alcoholic nor routine extraction methods [57] thus the study attempted to study an unusual method for extraction, and fortunately, it gave reasonable, considerable, valorized results.

### Gas chromatographic analyses

#### *Cichorium intybus*

**Fig. 2:**
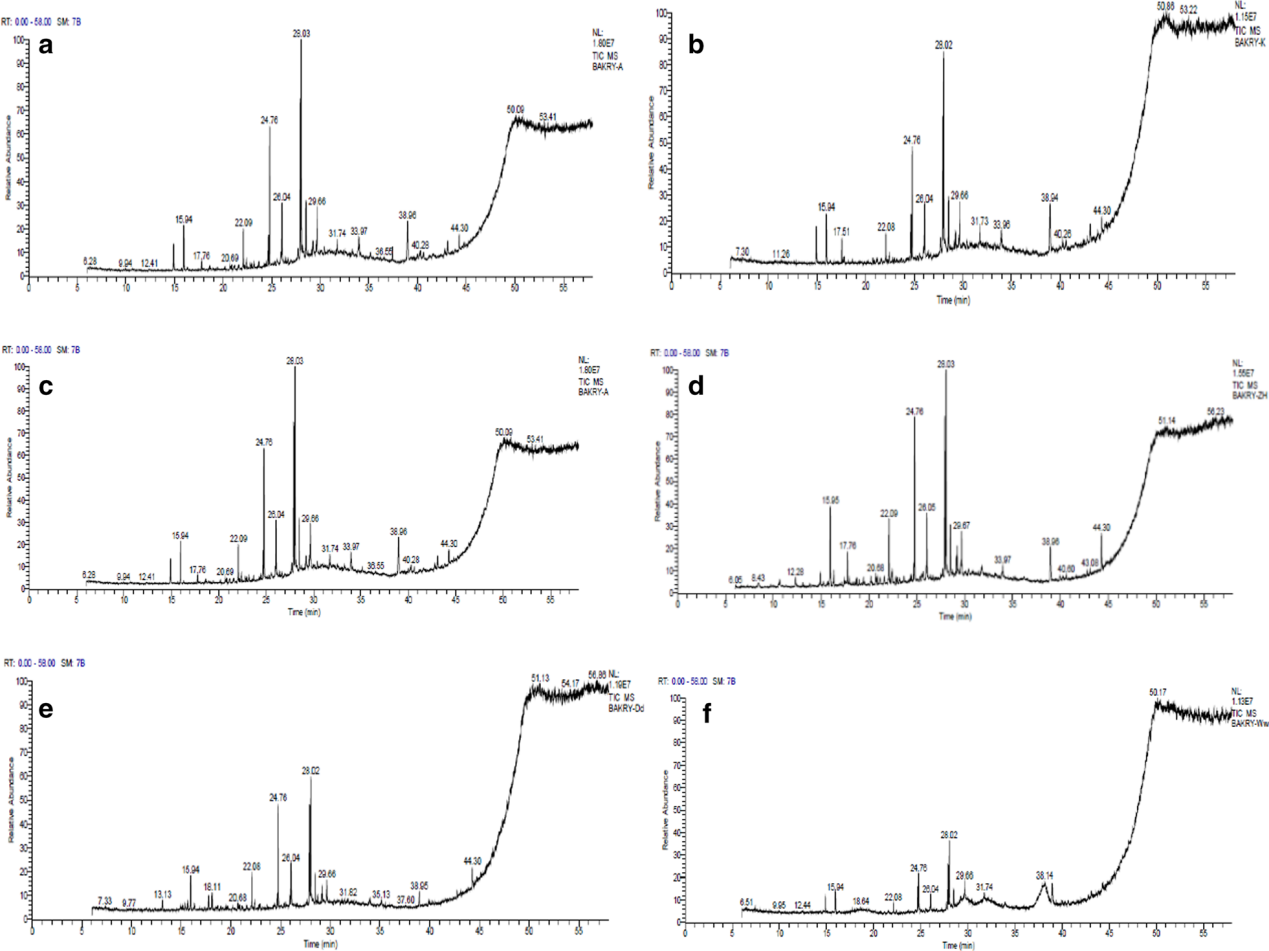
GC-MS chromatographs of SFE extracts of: **a***Cichorium intybus***b**; SFE *Cinnamomum camphora***c**; *Commiphora myrrha***d**; *Foeniculum vulgare***e**; *Nerium oleander***f**; *Spartium junceum*

There 13 variable compounds were detected in *C. intybus* by GC–MS analysis (Table 5 and Fig. 2a); most of them belong to fatty acid whether saturated or unsaturated as well as fatty acid precursors. Also, some phenolic and terpenoids were detected. Retinal is also known as retinaldehyde, which is a form of vitamin A produced by oxidation of retinol which functions as the active component of the visual cycle, this compound is unique for chicory.

**Table 5:** GC-MS report of SFE plant extracts exhibiting RT, molecular weight, molecular formula and frequency

Compound predicted	RT	M. wt	M. formula	1	2	3	4	5	6	F
Trans isoeugenol	12.28	164	C_10_H_12_O_2_				◙			1
Eugenol	16.31	164	C_10_H_12_O_2_				◙			1
Cyclohexane derivative	13.13	202	C_15_H_24_					◙		1
Dihydro butyl bezodoxepin	15.95	206	C_13_H_18_O_2_				●	●	●	3
Hydroquinone derivative	15.95	206	C_13_H_18_O_2_	●	●	●				3
Tetradecanol	17.76	214	C_14_H_30_O	●		●				2
Spathulenol	17.51	220	C_15_H_24_O		◙					1
Isofuranodionone	18.11	230	C_15_H_18_O					◙		1
Hexadecanol	17.76	242	C_16_H_34_O			●	●	●	●	4
Tridecanoic acid methyl ester	20.68	256	C_15_H_30_O_2_					◙		1
Tetradecanoic acid methyl ester	20.68	256	C_16_H_32_O_2_	●		●	●			3
Nonadecene	22.08	266	C_19_H_38_		●			●	●	3
Retinal	22.82	284	C_20_H_28_O	◙						1
Hexadecenoic acid methyl ester	24.76	270	C_17_H_34_O_2_	●	●	●	●	●	●	6
9-Eicosene	26.04	280	C_20_H_40_		●	●				2
Nonadecanoic acid	27.71	296	C_19_H_36_O_2_					◙		1
Octadecenoic acid methyl ester	28.03	296	C_19_H_36_O_2_	●	●	●	●	●	●	6
1-Eicosanol	26.05	298	C_20_H_42_O				◙			1
Heptadecanoic acid ethyl ester	26.05	298	C_19_H_38_O_2_	◙						1
Octadecanoic acid methyl ester	28.50	298	C_19_H_38_O_2_					●	●	2
Methyl stearate	28.50	298	C_19_H_38_O_2_		●	●	●			3
Linoleic acid ethyl ester	29.11	308	C_20_H_36_O_2_	◙						1
Eicosenoic acid	29.21	310	C_20_H_38_O_2_			●		●		2
Ethyl oleate	29.22	310	C_20_H_38_O_2_	●	●		●			3
Docosene	29.66	308	C_22_H_44_		●	●	●			3
Erucic acid	29.67	338	C_22_H_42_O_2_	●		●	●	●	●	5
Eicosenoic acid derivative	33.96	310	C_20_H_38_O_2_	◙						1
Behenyl alcohol or Doconasol	33.96	326	C_22_H_46_O		◙					1
Benzene dicarboxylic acid	38.95	390	C_24_H_38_O_4_	●	●	●	●	●	●	6
Docosanoic acid trihydroxy methyl ester	40.26	402	C_23_H_46_O_5_				◙			1
Squalene	44.30	410	C_30_H_50_				◙			1
Spirostenone	44.29	428	C_27_H_40_O_4_	●	●					2
Flavone dioglucoside	44.30	594	C_27_H_30_O_15_			◙				1
Total	33	33	33	13	12	13	15	13	8	

Numbers (1–6): 1; Chicory, 2; Camphor, 3; Myrrh, 4; Fennel, 5; Oleander, 6; Spanish broom. F means frequency, symbol ◙ means monomorphic peaks while symbol ● means polymorphic peaks

#### *Cinnamomum camphora*

Table 5 and Fig. 2b refer to predict the subcomponents of SFE *C. camphora* extract that point to the existence of 12 components belong to fatty acids and their precursors in addition to phenolics and terpenoids. Spathulenol is sesquiterpene alcohol as a unique compound for *C. camphora* during this study. On the other hand, Spirostenone belongs to terpenoids (isoprenoids), this compound found only in both *C.* intybus and *C. camphora*.

#### *Commiphora myrrha*

Table 5 and Fig. 2c expressed the prediction of subcomponents of SFE *C. myrrha* extract, analysis of GC–MS report displayed that 13 compounds had been detected; all these compounds belong to fatty acids and their precursors as well as phenolic and flavonoids.

#### *Foeniculum vulgare*

*Foeniculum vulgare* SFE extract was analyzed by GC–MS (Fig. 2d) to predict its subcomponents which were tabulated in Table 5 which revealed the detection of 15 subcomponents including fatty acids and their precursors. Eugenol and trans isoeugenol are unique for only *F. vulgare* which are classified as phenolic compounds, also squalene is alkene belong to isoprenoid compounds, this compound is also unique for *F. vulgare*.

#### *Nerium oleander*

*Nerium oleander* SFE extract was examined by GC–MS (Fig. 2e) to forecast its ingredients which were presented in Table 5 that shows the presence of 13 kinds of compounds including fatty acids and their precursors. Isofuranodionone is unique for *N. oleander* which is classified as a heterocyclic organic compound.

#### *Spartium junceum*

Only 8 compounds were detected in *S. junceum* SFE extract and this is the least content diversity among the examined plants, data was obtained by Fig. 2f that represents GC–MS chromatogram of *S. junceum* SFE extract, subsequently, this figure was analyzed to expect the ingredients which were arranged into Table 5 which showed a limited number and a limited diversity of subcomponents which belong to fatty acid (erucic acid) and precursors of fatty acids. GC–MS analyses for all examined plants showed the presence of fatty acids and fatty acids precursors in all investigated SFE extracts, although some compounds are unique for a specific plant among this study, on the other hands there are some compounds being common between two or more types of the plants under examination, for example; eicosanoic acid is monounsaturated (omega 9) fatty acid and it was detected in *C. intybus*, *C. myrrha*, and *N. oleander*. Erucic acid is also monounsaturated (omega 9) fatty acid and it was detected in *C. intybus*, *C. myrrha*, *F. vulgare*, *N. oleander*, and *S. junceum*. Doxepin derivative is an antidepressant molecule and it was detected in *F. vulgare*, *N. oleander*, and *S. junceum*. Spirostenone belongs to terpenoids (isoprenoids), this compound found only in both *C. intybus* and *C. camphora*. GC–MS report indicated the presence of residues of solvents involved in the extraction process plus the components constituting ethanolic clove extract [58] but fortunately, in this study, GC–MS report indicated the absence of the residues of solvents as an evidence to the high purity degree of the plant extract yielded by SFE.

## Conclusion

Most of the examined plants have medicinal importance especially as antimicrobial activity and those are rich in their phytochemical contents whether alkaloids, flavonoids, etc. which encourages the researchers to investigate more uncommon plants for their medicinal importance, not antimicrobial activities in particular. SFE equipment is an inspiring technique for plant extraction which offers time and effort saving as well as high purity of the crude extract with no organic residues in addition to superior results than the conventional extraction methods. There is a very crucial need for antimicrobial agents to be incorporated more and more into the pharmaceutical market especially those belong to natural sources to overcome the problem of microbial resistance, and the current study presented a primitive inspiring trial to help in complementary and alternative medicine. Also, a combination of those examples of natural antimicrobial agents and other established drugs may offer synergistic powerful effect in tackling the resistance problem. GC–MS reports displayed that some examined plants contain very useful unsaturated fatty acids (omega 9) and hence, those antimicrobial extracts may have an additional advantage or a dual-action.

## Data Availability

That all data and materials are available and included in the article.
